# Anther and ovule development of *Clematis serratifolia* (Ranunculaceae)*–*with new formation types in megaspore and nucellus

**DOI:** 10.1371/journal.pone.0240432

**Published:** 2020-10-15

**Authors:** Yi Yang, Jie Sun, Xiao Guo, Kuiling Wang, Qinghua Liu, Qingchao Liu

**Affiliations:** College of Landscape Architecture and Forestry, Qingdao Agricultural University, Qingdao, PR. China; Swedish University of Agricultural Sciences, SWEDEN

## Abstract

Morphological indices of vegetative organs or reproductive organs, which are often used to analyze the evolution and classify *Clematis*, indicate that *Clematis serratifolia* and *C*. *glauca* could be related members at similar evolutionary levels. However, this assumption differs with phylogenetic studies based on genetics. Embryonic characteristics, which are more stable, are commonly used to estimate the phylogeny and evolution of angiosperms. We studied the microsporogenesis, microgametogenesis, megasporogenesis and macrogametogenesis development of *C*. *serratifolia*, and compared the early embryological characteristics among *C*. *serratifolia*, *C*. *serratifolia* and other *Clematis* species reported to provide a reference for the taxonomy of the genus *Clematis*. Our results showed that *C*. *serratifolia* and *C*. *glauca* differ in megaspore formation and nucellus types suggesting that they have originated from different ancestors. The differences among *Clematis* were mainly found in the type of the anther wall development, tapetum, pollen grains, megaspore formation and nucellus types.

## Introduction

The genus *Clematis* belongs to the Ranunculaceae family, which is one of the ancestral groups of angiosperms, particularly, the eudicots [1]. It is a diverse genus of about 355 species with highly variable morphology and a wide distribution. The morphological indices of vegetative organs or reproductive organs of *Clematis* [2–5] have long been used as the basis for classifying *Clematis*. However, these indices have proved unstable in different environments. For instance, *C*. *pinnata*, *C*. *brevicaudata* and *C*. *heracleifolia* exhibit variable leaf morphology in different regions [6]. The classification of *Clematis*, therefore, has been controversial. In recent years, karyotype [7–9], pollen grain morphology [10] and molecular techniques [11–13] have been used to classify *Clematis*. However, the results are easily affected by the equipment and methods employed [7–9,14], and can be contradictory [11–13]. It is, therefore, necessary to find relatively stable morphological characteristics that can reflect the phylogeny and evolution to assist with the taxonomic study of *Clematis*.

Embryonic characteristics, which are commonly used to estimate the phylogeny and evolution of angiosperms [15,16], are relatively stable traits even in complex and changeable environments. Extensive embryological studies on Ranunculaceae have been carried out in recent years [17–22]. In *Clematis*, the anther development of *C*. ‘Ernest Markham’ [23] and *C*. *hexapetala* [24] have been studied, and the anther and ovule development of *C*. *fusca* [25], *C*. *terniflora* var. *mandshurica* [26], *C*. *heracleifolia* [27] and *C*. *glauca* [28] also have been observed in detail.

The morphological indices of *Clematis serratifolia* and *C*. *glauca* are similar. Therefore, they were considered to be related members at similar evolutionary levels [4]. However, this assumption differs from the findings of phylogenetic studies, which were based on ITS and chloroplasts DNA sequence [13]. Here, we studied the microsporogenesis, microgametogenesis, megasporogenesis and macrogametogenesis development of *C*. *serratifolia* to elucidate its early embryological characteristics. We analyzed the characteristics between *C*. *serratifolia* and *C*. *glauca*, and identified the similarities and differences in the early embryological characteristics of *Clematis*, to provide a reference for the taxonomy of *Clematis*.

## Materials and methods

*C*. *serratifolia* plants were grown from seed (collected from the natural site of Chimney Hill, Panshi city, Jiling, China) at Qingdao Agricultural University (36°20′N, 120°12′E, Qingdao city, Shandong, China) in April 2015. The mean annual temperature in this region was about 12.6°C.

Flower buds were collected every day from June to early September in 2018 and fixed in FAA (formalin: acetic acid: 50% ethanol, 1:1:18, v/v/v) immediately upon collection. Samples were embedded in paraffin by conventional methods and sliced by rotary microtome (Leica RM-2145, Shanghai Leica Instrument Co. Ltd., Shanghai, China). Ovary blocks were sectioned longitudinally with a thickness of 10 μm and anther blocks were sectioned transversely with a thickness of 8 μm. Sections were stained with Ehrlich’s hematoxylin and eosin [29], and examined using a light microscope (Leica DM 500, Shanghai Leica Instrument Co. Ltd., Shanghai, China).

After freeze-dring with tert-Butyl alcohol, pollen grains of *C*. *serratifolia* were coated with gold-palladium in a spatter coater (JFC-1600, JEOL, Japan) and observed under a scanning electron microscopy (2 kV, JSM-7500 F, JEOL, Japan). Characteristics of pollen grains, including the length of the polar axis (P), and the length of the equatorial axis (E), and spinule height and density were measured using Image J (National Institutes of Health, Bethesda, Maryland, United States of America).

## Results

### Microspore and microgametophyte development

**Fig 1:**
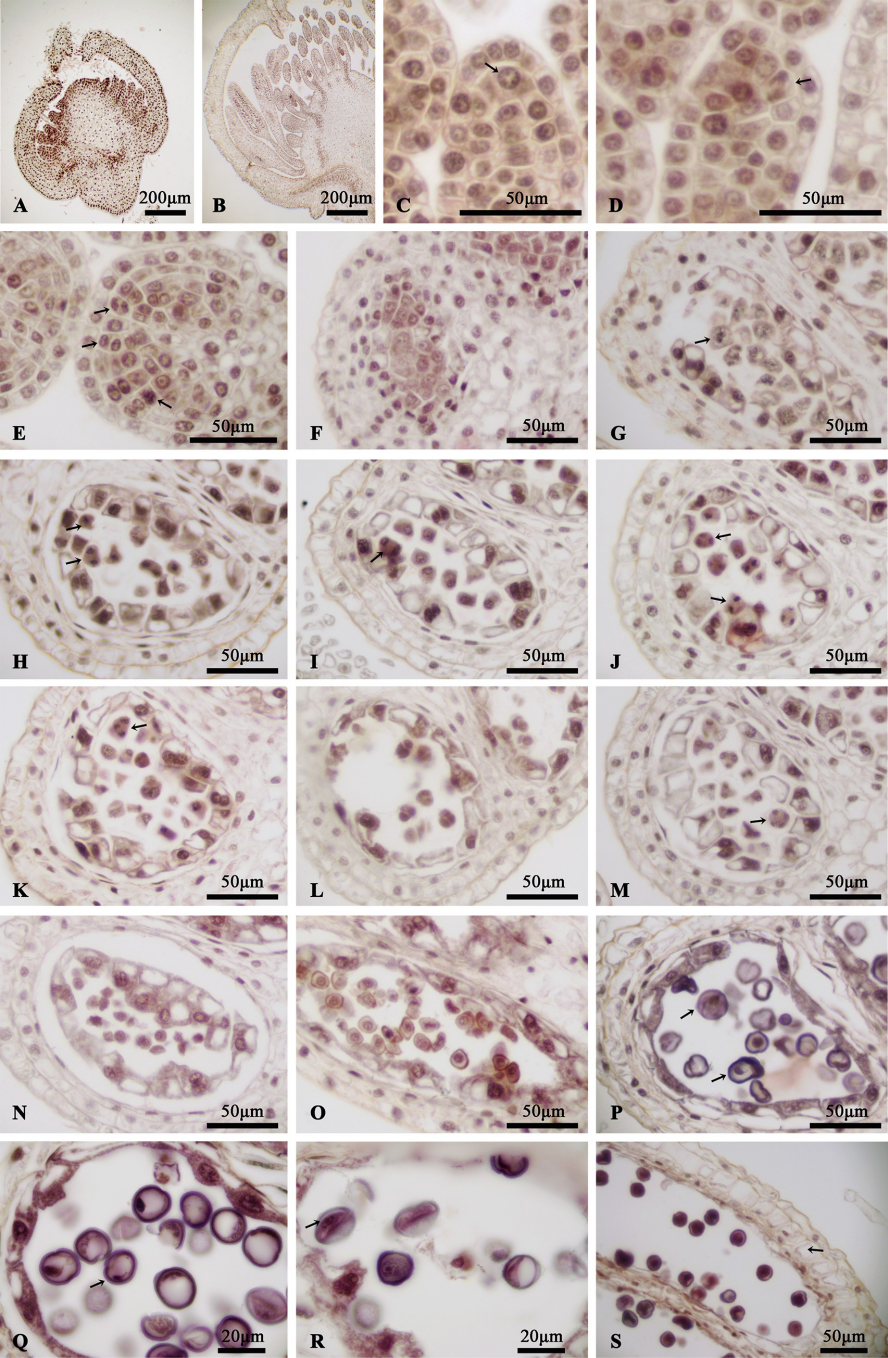
Microspore and microgametophyte development of *Clematis serratifolia*. (A-B) Flower bud differentiation; (C) Archesporial cell (arrow); (D) The archesporial cells divided into primary parietal cells and primary sporogenous cells (arrows); (E) Division of primary parietal cells (arrows, up and middle) and primary sporogenous cells (arrow, down); (F) A row of secondary sporogenous cell; (G) Microsporocyte cells at prophase of meiosis I (arrow); (H) Microsporocyte cells at metaphase (arrow, up) and anaphase of meiosis I (arrow, down); (I) Microsporocyte cells at telophase of meiosis I (arrow); (J) Microsporocyte cells at metaphase (arrow, down) and anaphase of meiosis II (arrow, up); (K) Microsporocyte cells at anaphase of meiosis II (arrows); (L) Microsporocyte cells at telophase of meiosis II (arrows); (M) Symmetrical microspores tetrads; (N) Disintegration of tetrad; (O) Monokaryotic centered stage microspore; (P) Monokaryotic side stage microspore (arrows); (Q) Microspore at metaphase; (R) Microspore at telophase, vegetative cell and germ cell are formed (arrow); (S) The mature anther structure, and the mature pollen with two-celled.

Early in June, the flower of *C*. *serratifolia* began to differentiate. The initiation and development patterns of floral organs were both centripetal (Fig 1A and 1B). After the development of perianth primordia, stamen primordia initiated and developed, and then matured gradually.

Stamen primordia were initially located under the epidermal cells and composed of actively dividing cells, from which the archesporial cells differentiated (Fig 1C). After the periclinal division of the archesporial cells, the outer primary parietal cells and inner primary sporogenous cells were formed (Fig 1D). The outer primary parietal cells then divided periclinally and anticlinally to form two layers of secondary parietal cells (Fig 1E), the outer layer of which divided again to form the endothecium and the middle layers, while the inter layer of secondary parietal cells developed directly into the tapetum. A mass of microspore mother cells were formed by several mitotic divisions of the primary sporogenous cells (Fig 1F).

The microspore mother cells then separated from each other and entered meiosis to form the tetrads (Fig 1G–1L). The tetrads were separated by the callose wall (Fig 1L and 1M), and the middle layers and tapetum cells gradually degenerated (Fig 1G–1M).

After the disintegration of the callose wall, the microspores were released from the tetrads (Fig 1N and 1O). With the expansion of its vacuoles, the microspore’s nucleus was displaced to one side of the endothecium (Fig 1P). Next, mitotic division of the microspore’s nucleus occured, resulting in a larger vegetative cell and a smaller generative cell (Fig 1P–1R). At this point, the middle layers and tapetum cells degenerated entirely, or left traces near the endothecium cells (Fig 1P–1R). Then, the radial and tangential walls of the endothecium were thickened, leading to anther dehiscence (Fig 1S). Until the shedding of the pollen grains, grains were two-celled monads.

### Pollen morphology

**Fig 2:**
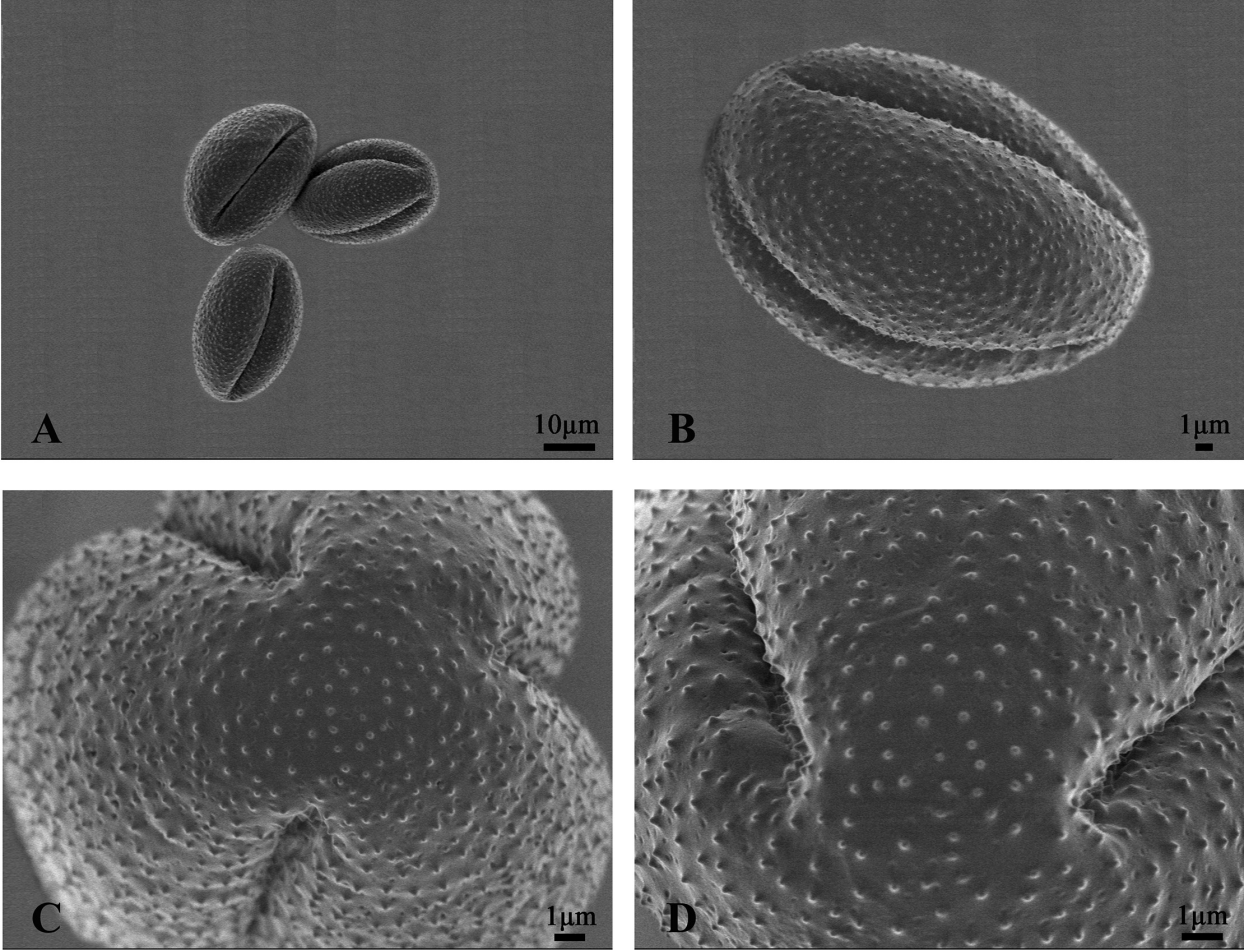
Scanning electron microscopy views of pollen grains of *Clematis serratifolia*. (A) The pollens of *Clematis serratifolia*; (B-C) Pollen in the equatorial view and in the polar view; (D) The ornamentation in pollen‘s surface.

The pollen of *C*. *serratifolia* was symmetrical monads, with three evenly distributed germ furrows (Fig 2A–2C). The outlines were trifid-round with an axis 20.66 μm in length in the polar view and an axis 30.57 μm in length in the equatorial view (Fig 2B and 2C; Table 1). On the pollen surface, perforation and microechinate ornamentation were clearly evident (Fig 2C and 2D).

**Table 1:** Pollen characteristics of *Clematis serratifolia*.

Characters	Data
Shape	prolate
Type of aperture	Tricolpate
E(μm)	30.57±0.87
P(μm)	20.66±0.88
P/E	1.48±0.06
Spinule height (μm)	0.20±0.03
Number of spinules (3×3 μm^2^)	11.30±3.14

Note: P means polar axis; E means equatorial axis; P/E means P/E ratio of pollen grains. n = 30.

### Megaspore and megagametophyte development

The ovary of *C*. *serratifolia* had one chamber with several ovules, of which only one ovule developed normally.

**Fig 3:**
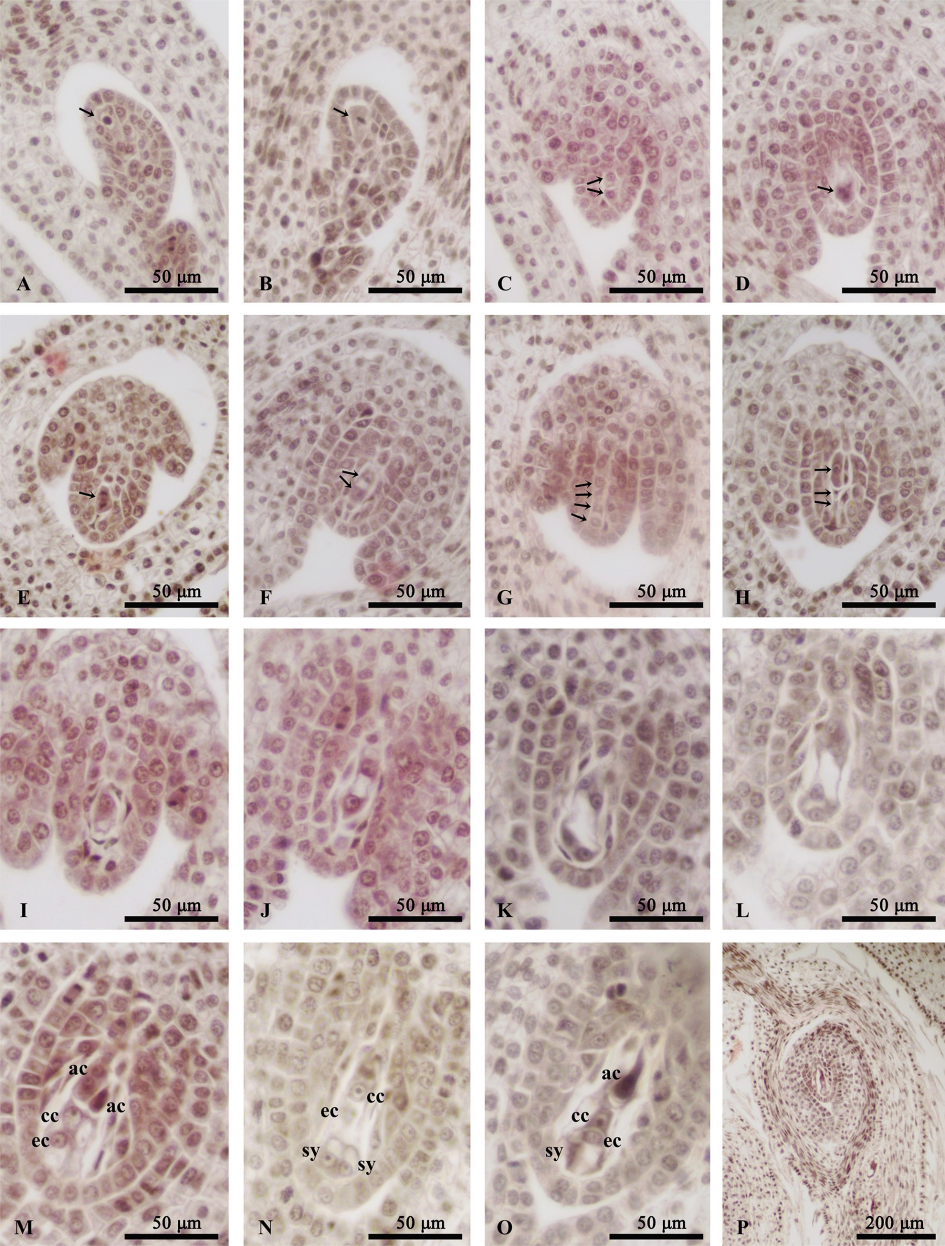
Megaspore and megagametophyte development of *Clematis serratifolia*. (A) Archesporial cell (arrow); (B) Archesporial cell at metaphase of mitosis; (C) The archesporial cell divided into a parietal cell (arrow, up), and a sporogenous cell (arrow, down), which formed into megaspore mother cell; (D-F) Megaspore mother cells at anaphase and telophase of meiosis I (arrows); (G) A linear tetrad of megaspores; (H) The three endostome megaspores degenerated(arrow, middle and down), and the chalazal one was functional megaspores(arrow, up); (I) The volume of functional megaspores increased; (J) Binucleate embryo sac; (K) Tetranucleate embryo sac; (L) Embryo sac with 8 free nucleis; (M-O) Mature embryo sac of seven-cells with eight nuclei; (P) Anatropous ovule. ac, antipodal cell; cc, central nucleus; ec, egg cell; sy, synergids.

Initially, a dome-shaped ovule primordium differentiated on the placenta epidermis, then formed a finger-like structure and developed into the nucellus. Subsequently, a single archesporial cell with large volume, dense cytoplasm and conspicuous nuclei appeared below the nucellus (Fig 3A). Two cells then formed after the periclinal division of the archesporial cell (Fig 3B and 3C). The outer (parietal) cell developed into the parietal tissue layer, and the inner (primary sporogenous) cell then directly differentiated into a megaspore mother cell (Fig 3D). At this point, the megaspore mother cell was surrounded by 2–3 layers of nucellus cells (Fig 3D). The ovules gradually curved (Fig 3D–3P), and a unitegmic integument formed (Fig 3D). Thus, the ovule of *C*. *serratifolia* was anatropous, unitegmic and crassinucellate.

A linear tetrad of megaspores was formed after the megasporocyte meiosis (Fig 3D–3G), with the chalazal megaspore developing normally and differentiating into the functional megaspore while the remaining megaspores becoming reduced (Fig 3H). In subsequent stages, the functional megaspore increased in volume (Fig 3I) and entered mitosis (Fig 3J–3O). It divided successively to form an embryo sac with 2 nuclei (Fig 3J), 4 nuclei (Fig 3K), and then 8 free nuclei (Fig 3L). The 8 free nuclei embryo sac entered cellularization and matured. The mature embryo sac (Fig 3M–3O) included three larger binucleated antipodal cells in the chalazal region, a diploid central cell, two synergids and an egg cell in the micropylar region. The embryo sac was similar to that found in *Polygonum*.

### Development relationship between gametophytes and flower bud morphology

In the same flower of *C*. *serratifolia*, the anther primordium developed before the pistil primordium. The female gametophytes developed slower than the male gametophytes, with a delay of about three to four stages at the beginning. However, the male and female gametophytes matured at approximately the same time before blooming. The developmental relationship between the gametophytes and the flower bud morphology were presented in Table 2.

**Table 2:** Development relationship between gametophytes and flower bud morphology of *Clematis serratifolia*.

Bud length (cm)	Stamen length (cm)	Pistil length (cm)	Male gametophy	Female gametophy
0.09–0.16	0.06–0.09	0.05–0.11	Archesporium,	_
0.16–0.27	0.09–0.13	0.11–0.18	Archesporium, microsporocyte	Archesporium
0.27–0.52	0.13–0.24	0.18–0.33	Microsporocyte, meiosis, monokaryotic	Archesporium, megasporocyte
0.52–0.72	0.24–0.33	0.33–0.50	Monokaryotic, monokaryotic side stage, mitosis	Megasporocyte, the linear tetrad, uninucleate embryo sac
0.72–0.98	0.33–0.42	0.50–0.62	Mitosis, two-celled pollen	Uninucleate, 2- nucleate, 4-nucleate, 8-nucleate or mature embryo sac
0.98–1.75	0.42–0.72	0.62–0.81	two-celled pollen	Mature embryo sac

## Discussion

### Microspore and microgametophyte development

We observed that the microspore and microgametophyte development of *C*. *serratifolia* and *C*. *glauca* were with only minor differences in the structure of the mature anther wall (Table 3). Features such as centripetal development of floral organs, tetrahedral microspores tetrads, simultaneous microsporocyte cytokinesis, and two-celled mature pollen grains may be the common anther development characteristics of *Clematis*. There were different in the type of anther wall development, tapetum, pollen grains and the structure of mature anther wall (Table 3).

**Table 3:** Early embryological characteristics of seven species in *Clematis*.

Genus	*Clematis serratifolia*	*Clematis glauca*	*Clematis terniflora* var. *mandshurica*	*Clematis heracleifolia*	*Clematis fusca*	*Clematis* ‘Ernest Markham’	*Clematis hexapetala*
Flower structure	Hermaphrodite	Hermaphrodite	Hermaphrodite	Androdioecy	Hermaphrodite	Hermaphrodite	Hermaphrodite
Development of floral organs	centripetal	centripetal	centripetal	centripetal	centripetal	centripetal	centripetal
Development of the anther wall	Monocotyledonous type	Monocotyledonous type	Monocotyledonous type	Basic type	Monocotyledonous type	Monocotyledonous type	Absent
Layers of the anther wall	4–5	4–5	4–5	4–5	4–5	4	4–5
Epidermis	Normal	Degenerate	Fibrous thickening	Fibrous thickening	Absent	Normal	Normal
Endothecium	Fibrous thickening	Fibrous thickening	Fibrous thickening	Fibrous thickening	Absent	Fibrous thickening	Fibrous thickening
Number of middle layer	1–2	1–2	1–2	1–2	1–2	1	1–2
Tapetum	Glandular	Glandular	Glandular, occasional amoeboid	Glandular, occasional amoeboid	Absent	Amoeboid	Glandular
Tetrad	Tetrahedral, occasional symmetrical	Tetrahedral, occasional symmetrical	Tetrahedral, occasional symmetrical	Tetrahedral, occasional symmetrical	Tetrahedral, occasional symmetrical	Tetrahedral, occasional symmetrical	Tetrahedral, occasional symmetrical
Microspore meiosis	Simultaneous	Simultaneous	Simultaneous	Simultaneous	Simultaneous	Simultaneous	Simultaneous
The mature pollen	two-celled, tricolpate	two-celled, tricolpate	two-celled, tricolpate	two-celled, pantocolpate	two-celled, tricolpate	two-celled, tricolpate	two-celled, tricolpate
Form of ovule	Anatropous	Anatropous	Anatropous	Anatropous	Anatropous	Absent	Absent
Integumentum	Unitegmic	Unitegmic	Unitegmic	Unitegmic	Unitegmic	Absent	Absent
Nucellus	Crassinucellate	Tenuinucellate	Tenuinucellate	Tenuinucellate	——	Absent	Absent
Archesporium	1	1	1	1	1	Absent	Absent
Differentiation of megaspores	Differentiated after the periclinal division of the archesporial cell	Differentiated directly from the archesporial cell	Differentiated directly from the archesporial cell	Differentiated directly from the archesporial cell	Absent	Absent	Absent
Tetrad of megaspores	Linear	Linear	Linear	Linear	Linear	Absent	Absent
The functional megaspore	Chalazal end	Chalazal end	Chalazal end	Chalazal end	Chalazal end	Absent	Absent
Embryo sac formation	*Polygonum*	*Polygonum*	*Polygonum*	*Polygonum*	*Polygonum*	Absent	Absent
Antipodal cells	Larger, dikaryotic	Larger, dikaryotic	Larger, dikaryotic	Larger, dikaryotic	Absent	Absent	Absent
Sources	Present study	[28]	[26,30]	[27,30]	[25]	[23]	[24,30]

Note: Absent means there is no data.——means there is a doubt.

It has previously been reported that the evolutionary trend in Ranunculaceae was from centripetal floral organs to centrifugal flower organs [21], from the amoeboid tapetum to glandular tapetum [31], from the basic type of anther wall development to monocotyledonous type [32], and from the tricolpate to the pantocolpate and the pantoporate [10]. Thus, *C*. *serratifolia*, along with the six species of *Clematis* (Table 3), showed a mix of ancestral and derived features.

### Megaspore and megagametophyte development

We observed that the megaspore and megagametophyte development of *C*. *serratifolia* and *C*. *glauca* were different in the megaspore formation and nucellus types (Table 3). Although the reported nucellus characteristics of *C*. *fusca* [25] were the same as *C*. *serratifolia*, evidence for this came from some fuzzy pictures and there was no conclusive information regarding the division of the archesporial cell and the nucellus type. It was, therefore, difficult to confirm the relevant characteristics of *C*. *fusca*, and we did not compare the ovule development characteristics of *C*. *fusca* with *C*. *serratifolia* here.

Studies have shown that the characteristics of megagametophyte development in Ranunculaceae were highly variable, including species with unitegmic integument (Tribe Helleboreae, Tribe Ranunculaceae and Tribe Anemoneae of Subfam. Ranunculoideae) or bitegmic integument (Subfam. Coptidoudeae and Subfam.Thalictroideae, Tribe Adonideae, Tribe Catheae, Tribe Nigelleae, Tribe Callianthemeae, Tribe Cimicifugeae and Tribe Delphineae of Subfam. Ranunculoideae), species with anatropous ovule (generally in Ranunculaceae) or hemianatropous ovule (*Adonis*, *Ranunculus* and *Batraehium*) [33], species with tenuinucellate, crassinucellate or both types of nucellus [18,34], species with linear, T-shaped tetrad of megaspores, or both [35,36], and species with uninucleate, dikaryotic or multinucleate antipodal cells [18,22]. There were also variations in the number of archesporial cells. Tamura considered that species in Ranunculaceae usually had only one archesporial cell, which can be observed in *Anemone*, *Aquilegia*, *Actaea*, *Adonis*, *Helleborus* and *Pulsatilla* [20]. However, species in *Caltha*, *Ranunculus*, *Delphinium* and *Cimicifuga* had 1–3 archesporial cells, and varieties of *Ranunculus septentrionalis* had 2–13 archesporial cells [20]. It can be seen that species of *Clematis* are anatropous and unitegmic, with a *Polygonum*-type embryo sac, a linear tetrad of megaspores, three large dikaryotic antipodal cells, and only one archesporial cell (Table 3). These may be common characteristics during the ovule development of *Clematis*. There were numerous convergences and differences in the early embryological characteristics between *Clematis* and other genera in Ranunculaceae.

Tamura considered that the megasporocyte of species in Ranunculaceae was usually formed by further division of the archesporial cell [37], as observed in *Trollius buddae* [20]. However, the single archesporial cell of *Helleborus thibetanus* [19] and *Caltha palustris* [20] developed directly into a megasporocyte, and the crassinucellate nucellus of the two species was derived from the division of the epidermis. It can be seen that the megasporocyte of *C*. *heracleifolia*, *C*. *glauca* and *C*. *terniflora* var. *mandshurica* all with tenuinucellate nucellus was formed directly by the archesporial cell, while that of *C*. *serratifolia* with crassinucellate nucellus was formed by further division of the single archesporial cell (Table 3). Thus, there were two megaspore formation types and two nucellus types in *Clematis*, and which of these types is more common needs to be studied further.

The characteristics of ovule development, especially integumentum, nucellus and ovule types, were of great reference value for species evolution and taxonomic studies. In dicotyledon, anatropous ovules, which were widely existed in the ancient plant groups such as Nymphaeaceae and Ranunculales, were generally considered as primitive characteristics [33,38–41]. Crassinucellate ovules commonly existed in the ancient plant groups, and bitegmic integument usually existed in the polypetalous, were both regarded as other primitive characteristics [41,42]. It can be seen that species of *Clematis* were commonly with ancestral features (anatropous ovules) and derived features (unitegmic integument). Studies have shown that species with crassinucellate ovules were usually with bitegmic integument [33,41]. However, there were two nucellus types in *Clematis*. The genus *Clematis* was overall not very original and its speciation process may be complex.

## Conclusion

Although *C*. *serratifolia* and *C*. *glauca* had similar morphological indices, there were differences in their early embryological characteristics, primarily in megaspore formation and nucellus types. Thus, *C*. *serratifolia* and *C*. *glauca* may have originated from different ancestors.

Common features of *Clematis* include centripetal development of floral organs, tetrahedral microspores tetrads, simultaneous microsporocyte cytokinesis, two-celled mature pollen grains, anatropous and unitegmic characteristics, *Polygonum*-type embryo-sac, a linear tetrad of megaspores, three larger dikaryotic antipodal cells and only one archesporial cell. The differences in early embryological characteristics among *Clematis* species were mainly found in the type of anther wall development, tapetum, pollen grains, megaspore formation and nucellus types.

There were numerous convergences in the early embryological characteristics within the species of the genus *Clematis*, or between *Clematis* and other genera in Ranunculaceae. The speciation process of *Clematis* was relatively complex, and interspecific hybridization could have occurred in its early differentiation.

## Supporting information

S1 Table Relevant data of pollen grains of *Clematis serratifolia*. (XLS)

S2 Table Relevant data of flower bud, stamen and pistil of *Clematis serratifolia*. (XLSX)
